# Assessing ecosystem service provision under climate change to support conservation and development planning in Myanmar

**DOI:** 10.1371/journal.pone.0184951

**Published:** 2017-09-21

**Authors:** Lisa Mandle, Stacie Wolny, Nirmal Bhagabati, Hanna Helsingen, Perrine Hamel, Ryan Bartlett, Adam Dixon, Radley Horton, Corey Lesk, Danielle Manley, Manishka De Mel, Daniel Bader, Sai Nay Won Myint, Win Myint, Myat Su Mon

**Affiliations:** 1 Natural Capital Project, Department of Biology and the Woods Institute for the Environment, Stanford University, Stanford, California, United States of America; 2 World Wildlife Fund, Washington DC, United States of America; 3 World Wide Fund for Nature, Yangon, Myanmar; 4 Department of Geography and Environmental Systems, University of Maryland, Baltimore County, Baltimore, Maryland, United States of America; 5 Center for Climate Systems Research, Earth Institute, Columbia University, New York, New York, United States of America; 6 NASA Goddard Institute for Space Studies, New York, New York, United States of America; 7 Forest Department, Ministry of Natural Resources and Environmental Conservation, Nay Pyi Taw, Myanmar; Centro de Investigacion Cientifica y de Educacion Superior de Ensenada Division de Fisica Aplicada, MEXICO

## Abstract

Inclusion of ecosystem services (ES) information into national-scale development and climate adaptation planning has yet to become common practice, despite demand from decision makers. Identifying where ES originate and to whom the benefits flow–under current and future climate conditions–is especially critical in rapidly developing countries, where the risk of ES loss is high. Here, using Myanmar as a case study, we assess where and how ecosystems provide key benefits to the country’s people and infrastructure. We model the supply of and demand for sediment retention, dry-season baseflows, flood risk reduction and coastal storm protection from multiple beneficiaries. We find that locations currently providing the greatest amount of services are likely to remain important under the range of climate conditions considered, demonstrating their importance in planning for climate resilience. Overlap between priority areas for ES provision and biodiversity conservation is higher than expected by chance overall, but the areas important for multiple ES are underrepresented in currently designated protected areas and Key Biodiversity Areas. Our results are contributing to development planning in Myanmar, and our approach could be extended to other contexts where there is demand for national-scale natural capital information to shape development plans and policies.

## Introduction

Attaining economic development goals while securing the natural capital and ecosystem service (ES) benefits that underpin current and future human well-being remains a major societal challenge. With the effects of climate change increasingly evident—and especially impacting developing countries–the challenge is even greater [1–3]. The adoption of the United Nations Sustainable Development Goals [4] and growing popularity of ‘green economy’ approaches to development [5] have been accompanied by an increasing demand from government decision-makers for ES information to guide development planning and climate change adaptation at national scales [6,7]. In addition, conservation organizations are increasingly incorporating ES into their programs in order to integrate conservation priorities into the broader socioeconomic context, to mainstream environmental issues into decision-making in the public and private sector beyond those institutions traditionally focused on the environment [8,9].

While the science and tools to support these efforts are advancing rapidly, substantial gaps remain between the promise of ES-based approaches and their application in common practice. ES assessments routinely focus on ecosystem properties and processes without considering whether and for whom actual benefits are produced [10]. Furthermore, consideration of the impacts of climate change on ES provision is rare, especially outside the U.S. and Europe [11]. Methodological differences among ES and climate assessments to date may pose additional challenges to integrated work. These gaps hinder the use of ES information for decision-making that is aimed at delivering durable gains in human well-being through an understanding of synergies and trade-offs between development activities and benefits from nature.

Here we provide a practical example from Myanmar to demonstrate how natural capital and climate change information can be integrated with data on the location and dependence of people and infrastructure on ES in order to contribute to development planning. Myanmar provides an excellent opportunity to advance natural capital assessment approaches and integrate their results into development policy and planning. After decades of restrictive military rule that both limited economic development and influenced the rate and nature of natural resource exploitation, the country now faces a unique crossroads: unlike so many countries that have prioritized economic development, but at great cost to their natural wealth, Myanmar may still have time to choose a path that allows for economic development while preserving ecosystems and the critical services they provide to the country’s citizens and its economy.

Myanmar’s development needs are immense: The country currently ranks 148 out of 188 in Human Development Index [12], and more than 25% of the population lives below the national poverty line [13]. Myanmar’s economy and people depend heavily on natural capital, with the livelihoods of over 70% of the population and nearly 40% of Gross Domestic Product reliant on agriculture, livestock, fisheries and forestry [14,15]. At the same time, the country is home to some of the largest remaining intact forests in Southeast Asia. However, the country lost more than 3% of its forest cover between 2000 and 2012, and the rate of deforestation has accelerated [16].

Improving understanding of Myanmar’s natural capital stocks and the ES benefits they provide is particularly relevant in the context of climate change. A recent report ranked Myanmar the second most historically vulnerable country to extreme weather events, due largely to the heavy toll of Cyclone Nargis in 2008, which killed upwards of 130,000 people in one of the deadliest tropical cyclones in history [17]. Extreme weather events seen in recent years–including coastal flooding associated with sea level rise and tropical cyclones, droughts, inland flooding and heat waves–are all projected to increase in frequency and intensity with climate change [18,19].

Recognizing the role of natural capital in the country’s prosperity and the security of its people, the Myanmar government is developing a number of strategies, procedures, rules and policies to improve environmental conservation in the country, including an overarching environmental policy aimed at mainstreaming environment and climate resilience into development planning and implementation [20]. Natural capital assessments have an important role to play in the implementation of Myanmar’s National Land Use Policy, which includes as one of its key objectives to “promote sustainable land use management and protection of cultural areas, environment, and natural resources for the interest of all people in the country” [21]. Similarly, Myanmar’s Climate Change Strategy and Action Plan is intended to enhance the resilience of the country to climate change [22].

In this context, there is a critical and immediate need for national-scale information on natural capital and the benefits it provides–both for local livelihoods and the larger economy–to contribute to planning the country’s national development pathway. Here we map and quantify how Myanmar’s natural capital provides key benefits to the country’s people and infrastructure, both under baseline climate conditions and a range of projected future climate conditions. This assessment resulted from a 2014 request by then-president of Myanmar, U Thein Sein, after seeing early outputs from this study at a sub-national scale. Specifically, we investigate at a national scale: 1) where natural ecosystems in Myanmar have the greatest potential to provide benefits in the form of four ES: sediment retention, regulation of dry-season water availability, flood risk reduction, and coastal storm protection; 2) where, given the locations of people and infrastructure that rely on these services, natural ecosystems are currently providing the greatest benefits; and 3) which beneficiaries are most susceptible to losing ES benefits from loss of natural vegetation.

To understand the degree to which these conclusions are sensitive to climate change, we assess whether the source and magnitude of benefits, as well as the susceptibility of beneficiaries, change under future climate scenarios. We use downscaled temperature and precipitation projections from 21 General Circulation Models (GCMs) [23], and regionalized sea level rise projections created using a blend of model- and observation-based terms [24,25]. To evaluate the complementarity between ES and other conservation objectives, we assess the degree to which the most important ES provision areas overlap with protected areas and Key Biodiversity Areas (KBAs; sites contributing significantly to the global persistence of biodiversity). Finally, reflecting on our experiences integrating ES and climate information in Myanmar, we suggest priority directions for a decision-relevant applied research agenda.

## Methods

### Myanmar context

**Fig 1:**
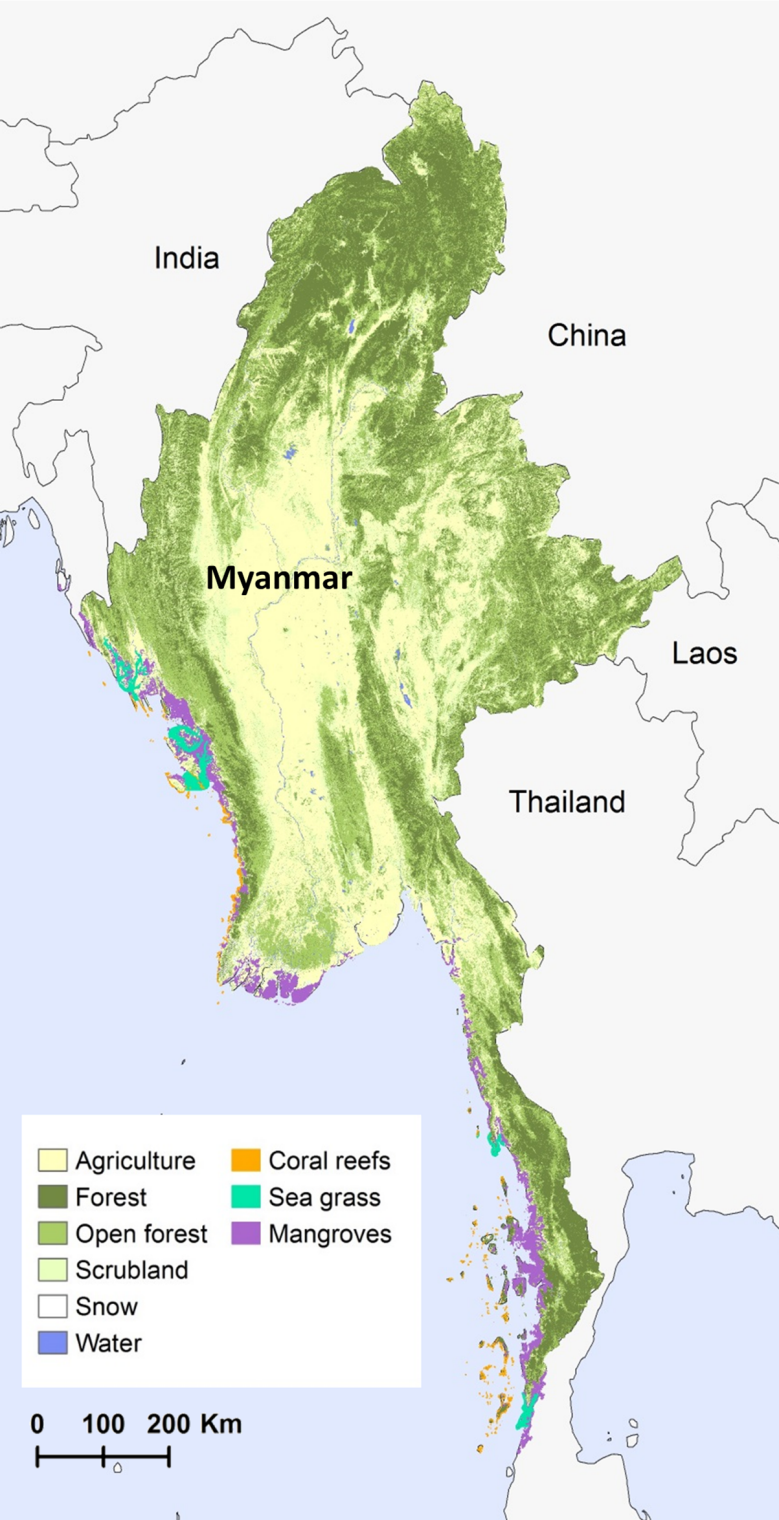
Myanmar’s land cover and coastal habitats. Less-visible land cover types include water bodies, which appear as large lakes and rivers through the central portion of the country, ending in the Ayeyarwady delta; and coral reefs, primarily located around islands in the most southern state of Tanintharyi and along the central Rakhine and Ayeyarwady coast. See S1 and S2 Appendices for details about the source and timeframe for each map component.

Myanmar is the largest country in mainland Southeast Asia. The country has a tropical monsoon climate, with three main seasons–cool (Nov.-Feb.), hot (Mar.-May) and wet (Jun.-Oct.). Myanmar contains a diversity of ecosystems (Fig 1), which harbor high levels of biodiversity, endemism and species richness. However, its natural resources are being degraded or lost as the country accelerates its pace of economic growth. With a population of 51.4 million, its per capita GDP is just over US$1,000, but GDP growth exceeds 8% [26].

In Myanmar, climate extremes have increased in frequency and intensity in recent decades, and are resulting in impacts like soil erosion and soil fertility loss, declining water availability and decreased river flows, inland flooding, and above-average storm surges, among many others [27]. These impacts are augmented by existing environmental stresses; high deforestation rates in Myanmar, for example, increase flooding and water quality impacts in areas downstream during extreme storm events as flood and soil retention services are compromised [27].

### Identifying ecosystem service provision areas

We focus on evaluating four ES: sediment retention for drinking water quality and reservoir function, regulation of dry-season baseflows for drinking water provision, inland flood risk reduction for flood-prone villages and coastal protection for coastal populations. These services were chosen based on their relevance in Myanmar to people, infrastructure and climate resilience, as well as on the availability of data and models required to evaluate them at a national scale.

We modeled the supply of these services under baseline climate conditions and under the bounds of a range of possible future climate conditions. We then integrated measures of biophysical supply with information on the location and number of beneficiaries to determine where ES provision is greatest, as well as to understand which beneficiaries would be most impacted by ES losses. Finally, we compared hotspots of ES provision with other, established priority areas for conservation–specifically, currently-protected areas and Key Biodiversity Areas–to determine where and the degree to which conservation has the potential to contribute to multiple objectives. We provide additional details about each step in the following sections.

### Modelling ecosystem service supply

To map and quantify the biophysical supply of the focal ES, we used the InVEST 3.2.0 Sediment Delivery Ratio (SDR), Seasonal Water Yield (SWY) and Coastal Vulnerability (CV) models [28] See S1 Appendix for more details about the models and data inputs. InVEST is a suite of quantitative, spatially explicit, production function-based ES models. Model outputs used in this study, described in detail below, are proxy variables that are interpreted in relative terms, rather than physical quantities. Additional information on model uncertainty and value interpretation is provided in the SI.

The SDR model estimates soil loss and sediment export in a watershed [29]. It relies on the widely-used Universal Soil Loss Equation (USLE) to compute soil loss based on climate, soil, topographic, and land use/land cover (LULC) information. The model converts soil loss estimates into sediment export, i.e. the amount of sediment observed in streams after retention by vegetation. In this study, we computed sediment retention maps as the difference in sediment export (expressed in tons/pixel) between the current LULC and a hypothetical extreme scenario where the current landscape was entirely converted to agriculture. In other words, this is the sediment retention benefit of maintaining the current vegetation. We chose agriculture as the alternative scenario for this heuristic experiment since agricultural expansion is an important driver of deforestation and is expected to increase as Myanmar’s agriculture sector becomes increasingly export oriented [30,31].

The SWY model estimates the amount of runoff production and relative contribution to baseflow of different parts of the landscape. Based on monthly precipitation, reference evapotranspiration, and LULC runoff properties, the model computes two indices representing quickflow and baseflow. Quickflow represents the amount of precipitation that is converted to direct runoff, entering streams soon after a rain event, and is computed based on the NRCS curve number method [32]. To assess flood risk reduction, we computed a non-dimensional flow retention index on each pixel as follows:

Flow retention=1-QF/P

where QF is the sum of quickflow over the main monsoon months (June–September) and P is total precipitation over those same months. The index ranges between 0 and 1; a value of 0 corresponds to no retention by a pixel, a value of 1 corresponds to total retention.

Baseflow represents the amount of precipitation that enters streams through subsurface flow, both during and between rain events, and is computed based on a local water balance and hydrologic routing. The index used in this study represents baseflow over the entire year. For both the flow retention and the baseflow indices, results are presented as the difference between the current landscape and the hypothetical alternate landscape in which all natural vegetation has been converted to agriculture.

The CV model produces a qualitative estimate of how changes to natural habitats can affect coastal communities’ exposure to storm-induced erosion and flooding [33]. By considering biological and geophysical factors along the coastline, the model differentiates areas with relatively high or low exposure to erosion and inundation during storms, and indicates the role that natural habitats play in helping reduce that exposure. Combining these results with global population information can show areas along a given coastline where humans are most vulnerable to storm waves and surge, and where natural habitats play the greatest role in protecting people. The output used from this model is “habitat role,” an index indicating how much protection coastal natural habitat provides to each shoreline segment. Habitat role is calculated as the difference in exposure to coastal storms between current habitat and a total loss of coastal habitats. This approach represents an extreme scenario of conversion but is useful for gauging relative importance of ecosystems at the national scale.

The current LULC map (Fig 1) used for the SDR and SWY models was based on a custom classification of 2013–2014 Landsat imagery (S2 Appendix). Areas classified as agriculture were further differentiated by the types of crops grown in each administrative district, using information from the FAO Digital Agricultural Atlas of the Union of Myanmar [34]. In general, across the whole country, rice is the dominant crop, except in the interior east. Pulses are also commonly grown, particularly in the interior east, and this area also is a significant producer of sesame. Along with rice, maize is an important crop of the northeast, while oil palm and rubber make up a significant percentage of agriculture in the south.

For the hypothetical scenario in which natural vegetation is converted to agriculture, all LULC classes not already included in agriculture/urban/bare were changed to agriculture, assuming the district-specific mix of crops. See S1 Appendix for more details. The locations of coral reefs, sea grass and mangroves (Fig 1) used to assess the role of these habitats in reducing coastal vulnerability were based on data from UNEP-WCMC [35–37]. Continental mangrove data was updated for the Tanintharyi region with data provided by Myanmar’s Ministry of Environmental Conservation and Forests (now the Ministry of Natural Resources and Environmental Conservation), created in 2015.

### Climate scenarios and integration into ecosystem service models

We used downscaled estimates of temperature, precipitation and sea level under baseline climate conditions (1980–2005 for temperature and precipitation, 2000–2004 for sea level rise) as well as projections of future climate conditions. To represent the uncertainty in climate projections, we used the bounds of a range of future climate conditions, rather than a single average estimate.

Baseline and future climate conditions for temperature and precipitation were developed from the NASA Earth Exchange Global Daily Downscaled Projections (NEX-GDDP) dataset (NASA, 2015). The resolution of both the observed and the projected products is .25 degrees by .25 degrees. For temperature and precipitation (total amount and number of rainfall events > 0.2 mm), monthly means of long-term average climatology were projected for the 2040 (2031–2050) time period for each 0.25 degree gridcell and calendar month. The combination of 21 climate models and two climate forcing scenarios, or Representative Concentration Pathways (RCPs) [38], yielded 42 potential outcomes for the projected time period. For each gridcell and calendar month, the 25^th^ percentile of numerically ranked model results for RCP 4.5 was defined as a low estimate for changes in climate, while the 75^th^ percentile of numerically ranked model results for RCP 8.5 was defined as a high estimate. These points in the model output distribution represent a range of likely future climates. Because the 25^th^ and 75^th^ percentiles are defined separately for each calendar month and gridcell, different GCMs can occupy the 25^th^ and 75^th^ percentiles in different months and locations. This approach broadens the range of possible outcomes compared to selecting a single GCM based on the spatially and temporally averaged 25^th^ and 75^th^ percentiles of the distribution.

Sea-level rise was projected for the 2050s (2050–2059) time period. Twenty-four models were used from Coupled Model Intercomparison Project 5 (CMIP5) to account for thermal expansion of the ocean and dynamically-driven changes in relative ocean height, with other data sources and methods accounting for land-based ice loss and changes in land water storage [24,25]. Due to the lack of long-duration tide gauge data in Myanmar, local land subsidence or uplift could not be accounted for in the sea level rise projections. The low estimate for sea level rise was derived from the 25^th^ percentile of the combined RCP 4.5 and 8.5 numerically ranked model outcomes, and the high estimate was derived from the 75^th^ percentile of the combined numerically ranked RCP 4.5 and 8.5 outcomes. The spatial resolution of the sea level rise was 1.0 degree.

The low and high ends of the range of climate change estimates, along with baseline climate, were translated into inputs for the InVEST models. See S1 Appendix for details. The SDR model includes climate in the form of annual average rainfall erosivity, which we calculated based on annual precipitation amount summed from the monthly projections. The SWY model includes precipitation amount, number of rainfall events and reference evapotranspiration all at the monthly time scale. Reference evapotranspiration was calculated from precipitation amount and temperature using the Modified Hargreaves method [39]. Both precipitation amount and number of rainfall events were obtained directly from the baseline or projected climate data. We modeled SWY under the low and high ends of the climate range using either all low or all high estimates of precipitation amount, number of rainfall events or temperature. Sea-level rise projections were used in the CV model. All climate estimates were used at their native resolution. To calculate ES supply and benefits under climate change, we again compared the current landscape with a hypothetical alternate landscape (conversion to agriculture for SDR and SWY models; complete loss of mangroves, seagrasses and marshes for the CV model).

There are a few limitations that need to be considered when interpreting the climate information and projections. First, the combination of limited historical climate data and a highly spatially heterogeneous climate associated with complex topography and coastlines may in turn affect the skill of the climate projections, which by necessity given the data shortages are based upon a modeled representation of baseline climate (and therefore subject to any limitations within the NEX-GDDP dataset). Second, while the projections presented here are designed to capture a range of possible outcomes, as with all climate projections, deep uncertainties render it impossible to develop truly probabilistic projections Finally, these climate scenarios were not translated into climate change-induced projected changes in LULC; further changes in ES provision are likely depending on future land use change, driven by the direct response of vegetation to climate change or human responses to shifting rainfall and temperature increases, but were not modeled due to a lack of national-scale information at sufficient resolution.

### Beneficiaries and linking ecosystem service supply to benefits

We used data on the location and needs of people and infrastructure to assess the provision of ES benefits. We considered two benefits from sediment retention: drinking water purification and reservoir function. In the case of drinking water purification, the beneficiaries included households reliant on surface water for drinking. We determined the number of households per township using surface water for drinking based on the 2014 Myanmar Population and Housing Census [40]. Because some areas were not enumerated in the 2014 census, our results underestimate the level of service provision in these parts of the country, particularly Kachin, Rakhine and eastern Shan states. In the case of reservoir function, we considered reservoirs as beneficiaries and used the GRanD database [41] to determine the location of these facilities. While this database omits many smaller reservoirs in Myanmar, no additional information on reservoir location was available at the time of analysis. Therefore, our results provide a conservative estimate of sediment retention services for reservoir function.

For inland flood risk reduction, we focused on villages that were affected by flooding in 2015 [42], with the assumption that further loss of this ES benefit would increase flood risk to these already-susceptible populations. In the case of dry-season water availability, we considered households relying on surface water for drinking as beneficiaries, using the same 2014 township-level census data as for drinking water quality.

We used a serviceshed-based approach to spatially link ES supply to beneficiaries [43,44]. A serviceshed is the area that provides a specific ES to a particular beneficiary. We used the InVEST tool DelineateIt [28] to map the watersheds contributing surface water to towns, villages and dams. These watersheds served as servicesheds for water purification and dry-season water availability, based on the assumption that people relying on surface water use nearby water bodies in the case of drinking water-related services. In the case of flood-affected villages, we assumed these same watersheds constituted the servicesheds that influenced inland flood risk. For coastal vulnerability, we considered the total population living within 3 km of a focal shoreline segment as beneficiaries, calculated from population density data from WorldPop [45].

We translated the biophysical metrics of ES supply from the InVEST models into importance for ES provision by weighting supply by the number of beneficiaries receiving a particular service from a particular place [7,44]. For drinking water quality and dry-season water availability, we multiplied supply by the number of surface water-dependent households located in all downstream watersheds. Because the SDR model outputs estimates of sediment loads (in tons), whereas drinking water quality is generally more affected by sediment concentration, the supply of sediment retention for drinking water quality was standardized by the catchment size serving surface water-dependent households. We therefore use catchment area as a proxy for runoff volume to compare sediment concentration across catchments, rather than sediment loads. In the case of flood risk reduction, we weighted supply by the number of downstream villages, as household counts were not available at this resolution. For coastal vulnerability, we weighted supply by the total population located within 3 km of the focal habitat.

To understand the susceptibility of townships to the loss of ES benefits, we calculated the percent change in sediment loads, dry-season baseflows, inland flood risk index and coastal vulnerability between the current landscape and the alternate land use scenario, for both baseline climate conditions and under the range of climate change estimates.

### Overlap between priority biodiversity conservation and ecosystem service provision areas

To understand the degree to which areas important for providing the focal ES coincide with biodiversity conservation priorities, we examined the overlap between Key Biodiversity Areas (KBAs) [46], protected areas (PAs) [47], and hotspots of ES provision in Myanmar. KBAs are sites contributing significantly to the global persistence of biodiversity [48]. Shapefiles for KBAs in Myanmar were obtained from BirdLife International *et al*. [49]. We defined ES hotspots as the top 20% of service provision area for each of three freshwater services: sediment retention for drinking water quality, dry-season baseflow for drinking water availability and inland flood risk reduction for flood-prone villages. We combined these to identify which areas were hotspots for one, two or all three of these services, and overlaid these with maps of terrestrial KBAs (making up 16.3% of Myanmar’s area) and protected areas (PAs, making up 7.4% of Myanmar’s land area) and determined the amount of overlap. The observed area of overlap was determined using ArcGIS. We compared observed overlap with the amount expected by chance based on the joint probability distribution assuming independence, both among hotspots for different services and between ES hotspots and biodiversity priority areas (KBAs and PAs). The probability of multiple independent events co-occurring is the product of the probabilities of each event occurring separately. Expected overlap by chance was calculated in this way using Excel, based on the fraction of Myanmar’s land area covered by each ES hotspot (by definition, 0.2), as well as by KBAs (0.163) and PAs (0.074).

## Results

### National-scale ecosystem service supply and provision under current conditions

**Fig 2:**
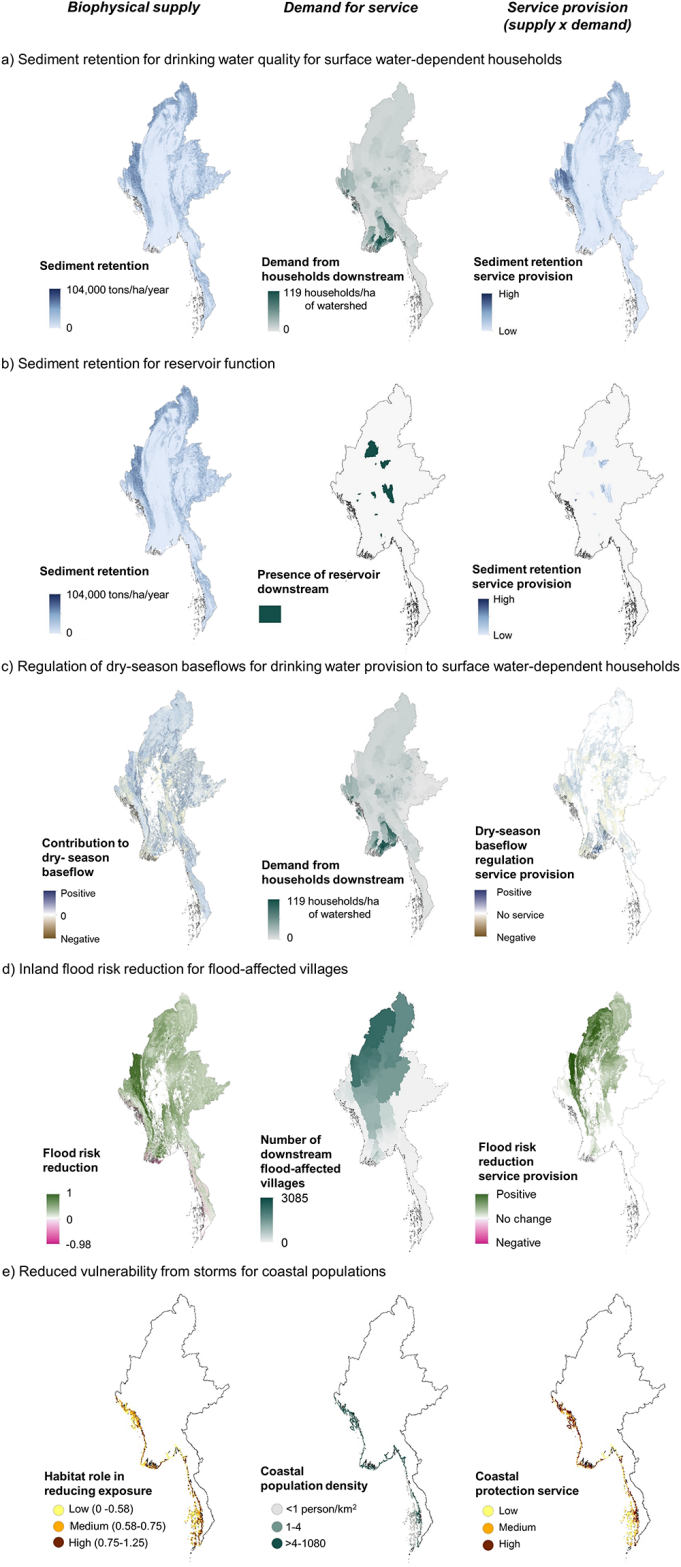
Ecosystem service supply, demand and service provision under baseline climate conditions. Biophysical supply, or potential for ES provision (left), demand for ES from beneficiaries (center) and service provision (supply x demand, right) for: a) sediment retention for drinking water quality, b) sediment retention for reservoir function, c) dry-season baseflows for drinking water availability, d) inland flood risk reduction for flood-affected villages, and e) coastal protection for coastal populations. Sediment retention is expressed as the benefit of maintaining the current vegetation over agricultural development, see Methods for details.

The biophysical supply of ES, indicating the potential to provide ES benefits, varies within Myanmar and along its coastline as a function of climate and topography, along with properties of the current vegetation and the assumed alternative land use (Fig 2, left column). For example, natural vegetation contributes most to sediment retention in currently forested areas situated on steep slopes, especially near to streams. Not only is soil loss higher in the areas converted to agriculture, but it is also less likely to be retained by the degraded landscape downslope before reaching the stream. Natural vegetation increases dry-season baseflows in areas with dense forest, where deep-rooted trees contribute to higher infiltration rate. Natural vegetation plays the greatest role in reducing inland flood risk in places with dense forest and high precipitation, where peak flow is higher. Mangroves are very effective at reducing coastal vulnerability to storms, especially compared to seagrass beds [50], and so the presence of mangroves (Fig 1) generally corresponded with the highest role of coastal habitat in reducing exposure (Fig 2e).

Demand for service also varies spatially, depending on the nature of the service and the beneficiaries considered (Fig 2, middle column). Demand for services linked to drinking water quality (sediment retention, Fig 2a) and seasonal availability (dry-season baseflows, Fig 2c) is concentrated in the watersheds that supply urban population centers towards the south. In contrast, for the 15 reservoirs assessed, demand for sediment retention is restricted to the specific watersheds serving each dam. There is demand for inland flood risk reduction services (Fig 2d) especially from watersheds in the north that regulate flows to villages that were affected by flooding in 2015. Demand for reduced vulnerability to coastal storms (Fig 2e) varies with the density of coastal populations.

Patterns of service provision, indicating the areas of natural vegetation that provide the greatest biophysical amount of service to the greatest number of beneficiaries, depend on both the location of supply and demand for each service and are unique for each service considered (Fig 2, right column).

**Fig 3:**
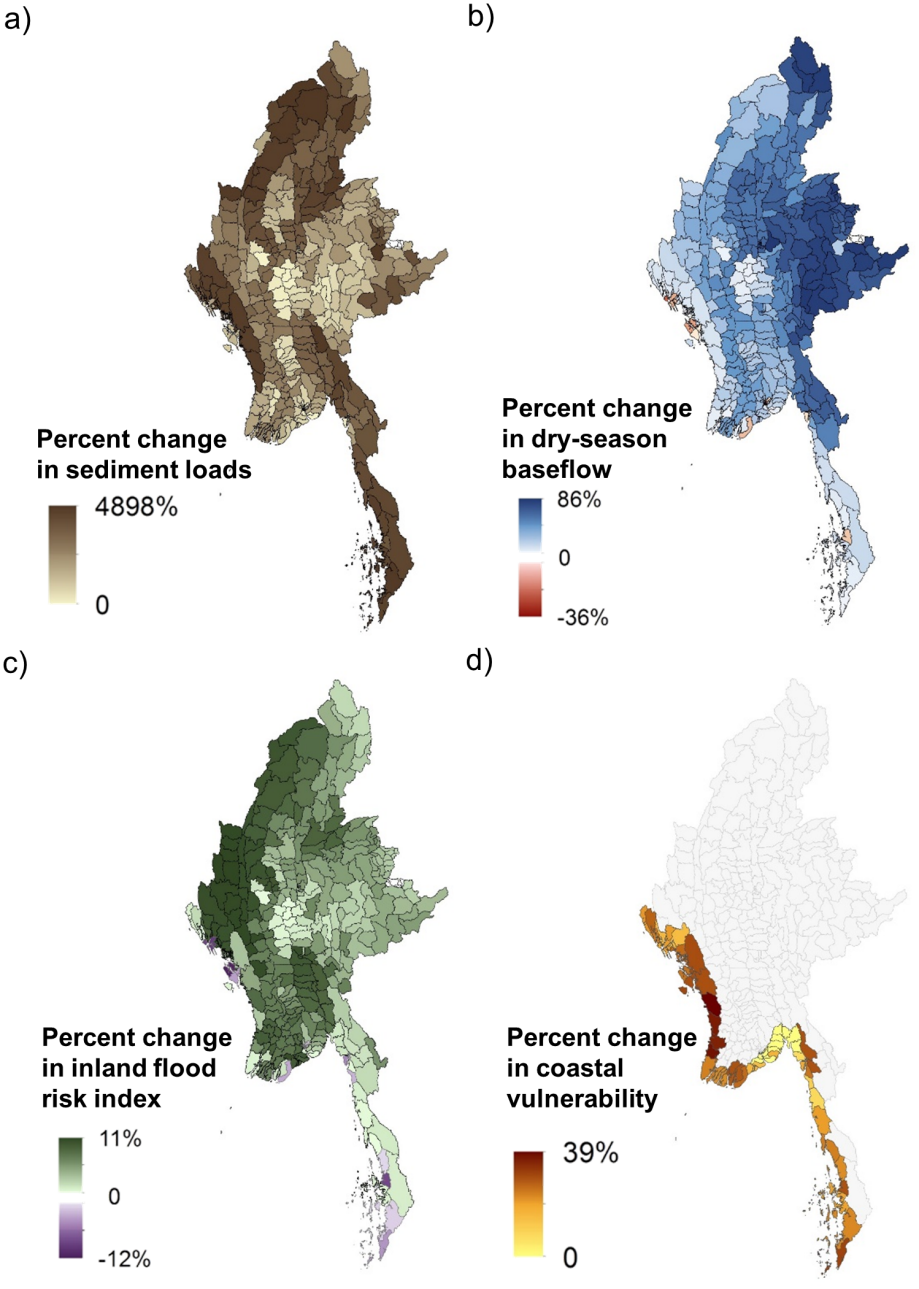
Susceptibility of townships to loss of ES from loss of natural vegetation under baseline climate conditions. a) sediment retention for drinking water quality, b) dry-season baseflow regulation for water availability, c) inland flood risk reduction, and d) coastal protection. For coastal protection, townships without coastal areas are shown in gray.

The degree to which people are susceptible to reductions in drinking water quality and dry-season water availability, as well as to increases in inland flood risk and vulnerability to coastal storms also varies spatially and by service (Fig 3). Townships in mountainous areas in the north, west and south would experience the greatest increase in sediment loads in drinking water sources with loss of natural vegetation. In contrast, loss of natural vegetation would lead to the greatest reduction in dry-season baseflows for townships near Myanmar’s eastern border, and to the greatest increase in inland flood risk index in the west. Townships in the southern portion of the Dawei peninsula and in southern Rakhine state to the north would experience the greatest increases in coastal vulnerability with the loss of coastal habitats.

### Effect of climate change scenarios on ecosystem service provision

For all GCMs, calendar months and Myanmar regions, temperatures are projected to rise as the century progresses, with the largest warming associated with RCP 8.5, which corresponds to current global emissions trends. In general, slightly more warming is projected in interior regions than in southerly and coastal areas. Precipitation is projected to increase in most, but not all GCMs. In general, precipitation increases are projected to be largest in the monsoon season. See Supporting information (S2–S4 Data) and Horton et al. [51]for more detail on the range of projected changes in temperature and precipitation. Changes in average ocean height are expected to be essentially homogenous across the entire Myanmar coast and by season. As noted in the Methods, changes in the elevation of the land itself will be heterogeneous with deltas especially prone to land subsidence; however, land elevation changes were excluded from this analysis due to the absence of tide gauge data.

**Fig 4:**
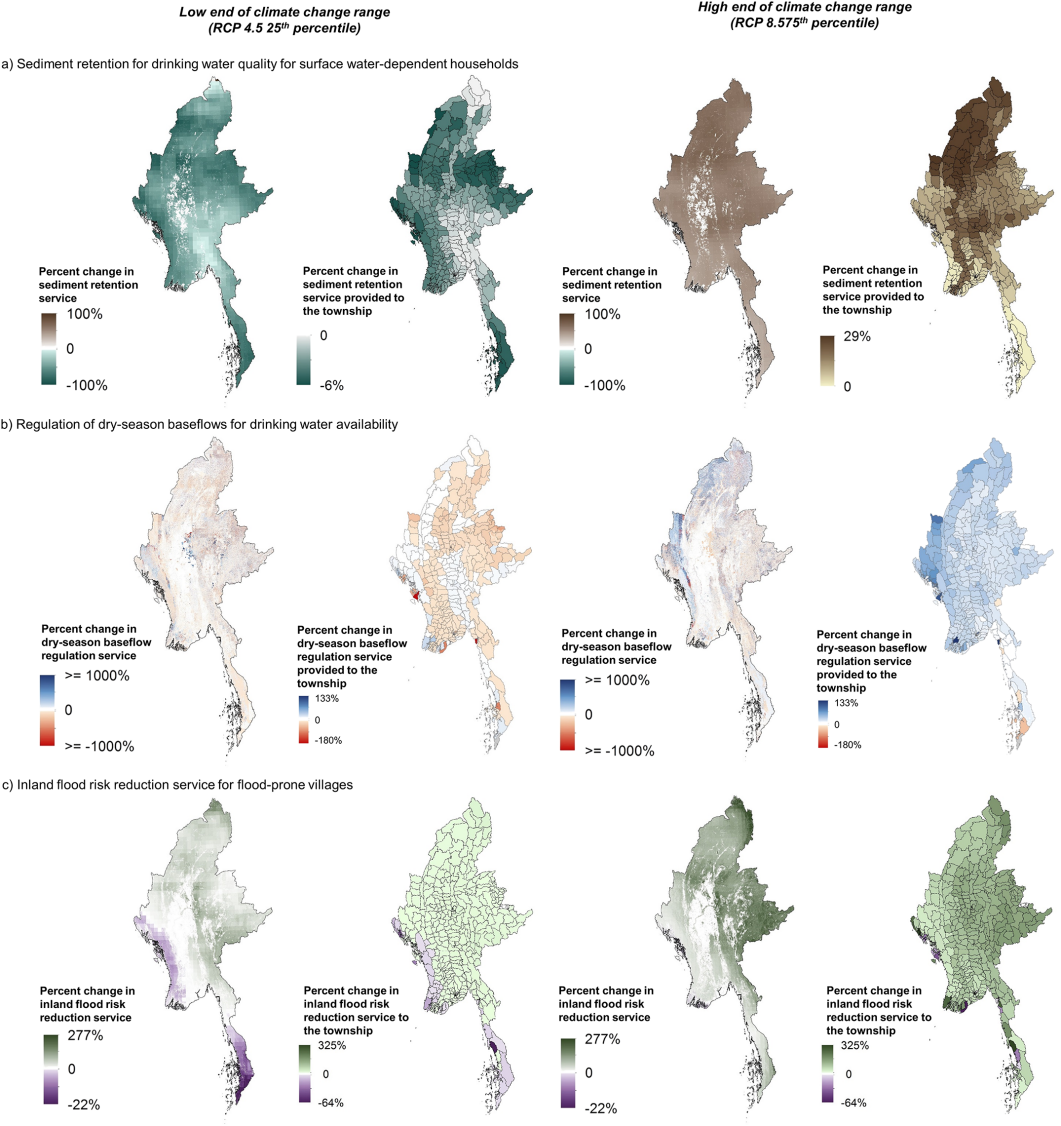
Change in ES supply and susceptibility of townships to ES losses from loss of natural vegetation in the 2040s with climate change relative to baseline climate conditions. Change is shown across three freshwater services: a) sediment retention for drinking water quality, b) regulation of dry-season baseflows for drinking water availability, and c) inland flood risk reduction for flood-prone villages. For each climate change scenario, change in ES supply is shown in the left column and change in susceptibility to loss is shown in the right column. Susceptibility to loss is measured as the percent change in service with loss of natural vegetation between the climate change scenario for the 2040s and historic climate conditions.

Projected changes in climate are expected to alter ES provision in multiple ways. Under the high estimate for changes in climate showing increasing precipitation, natural ecosystems are expected to play even greater roles in retaining sediment, regulating dry-season water availability and reducing inland flood risk (Fig 4, third column), making the populations who depend on these services even more vulnerable to the loss of natural ecosystems (Fig 4, fourth column). At the low end of the range of projected future rainfall, in which annual precipitation declines slightly even as variability and intensity of the strongest rainfall events likely increases, supply of some services may be reduced in certain areas. This is particularly the case for sediment retention and for dry-season baseflow regulation (Fig 4, first and second columns). For sediment retention, reduced precipitation lessens expected erosion with loss of natural vegetation, resulting in reduced service provision (median = -4% reduction in avoided sediment loss as compared to baseline climate conditions), while increased precipitation under the high estimate leads to increased erosion with loss of natural vegetation and therefore increases the service provided by retaining natural vegetation (median = 23% increase in avoided sediment loss).

Precipitation changes are likely to also alter both the amount of sediment reaching reservoirs and being retained by upstream vegetation. For the 15 reservoirs assessed in this study, sediment export to reservoirs in the absence of any land use change was estimated to decrease by an average of 3.4% (standard deviation (SD) = 1.2%) at the low end, but increase by 23.3% (SD = 3.3%) at the high end of the range of likely climate change. In addition, the amount of sediment retained upstream decreased an average of 3.4% (SD = 1.2%) under the low estimate and increased 23.3% (SD = 3.4%) under the high estimate.

Because the CV model used to assess coastal protection services is a relative ranking model and projected SLR was consistent along Myanmar’s coastline, the relative importance of coastal habitats did not change between SLR scenarios relative to baseline climate conditions (Fig 2e), and so these results are not shown separately here.

**Fig 5:**
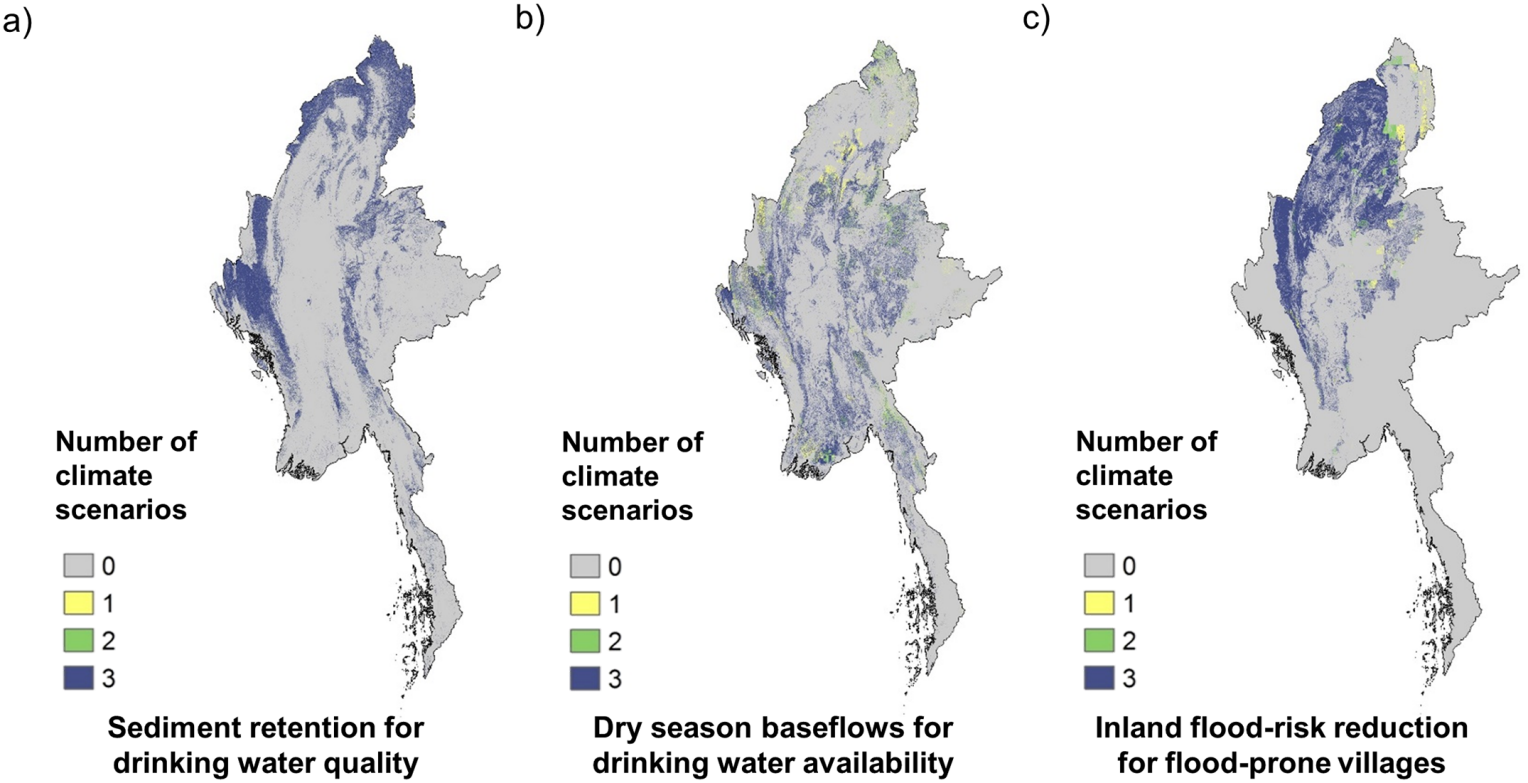
Concordance in areas providing the greatest levels of ES across historic climate conditions and the low and high ends of the projected climate range in the 2040s. a) sediment retention for drinking water quality, b) regulation of dry-season baseflows for drinking water availability, and c) inland flood risk reduction for flood-prone villages. The area is shaded according to whether it falls in the top 20% of service provision areas for a given service under one, two or three of the climate scenarios considered.

Regardless of climate change scenario, the parts of the landscape that currently provide the greatest amounts of services under baseline climate conditions are likely to remain important under the climate change scenarios considered here (Fig 5). We considered hotspots as the area that fell within the top 20% of provision for a given service and climate scenario (baseline, low estimate for climate change, high estimate for climate change). Hotspot areareas were consistently classified as hotspots across all three climate scenarios in 95% of instances for sediment retention for drinking water quality, 79% of instances for dry-season baseflows for drinking water availability, and 92% of instances for inland flood risk reduction.

### Spatial overlap of conservation priority areas at a national scale

**Fig 6:**
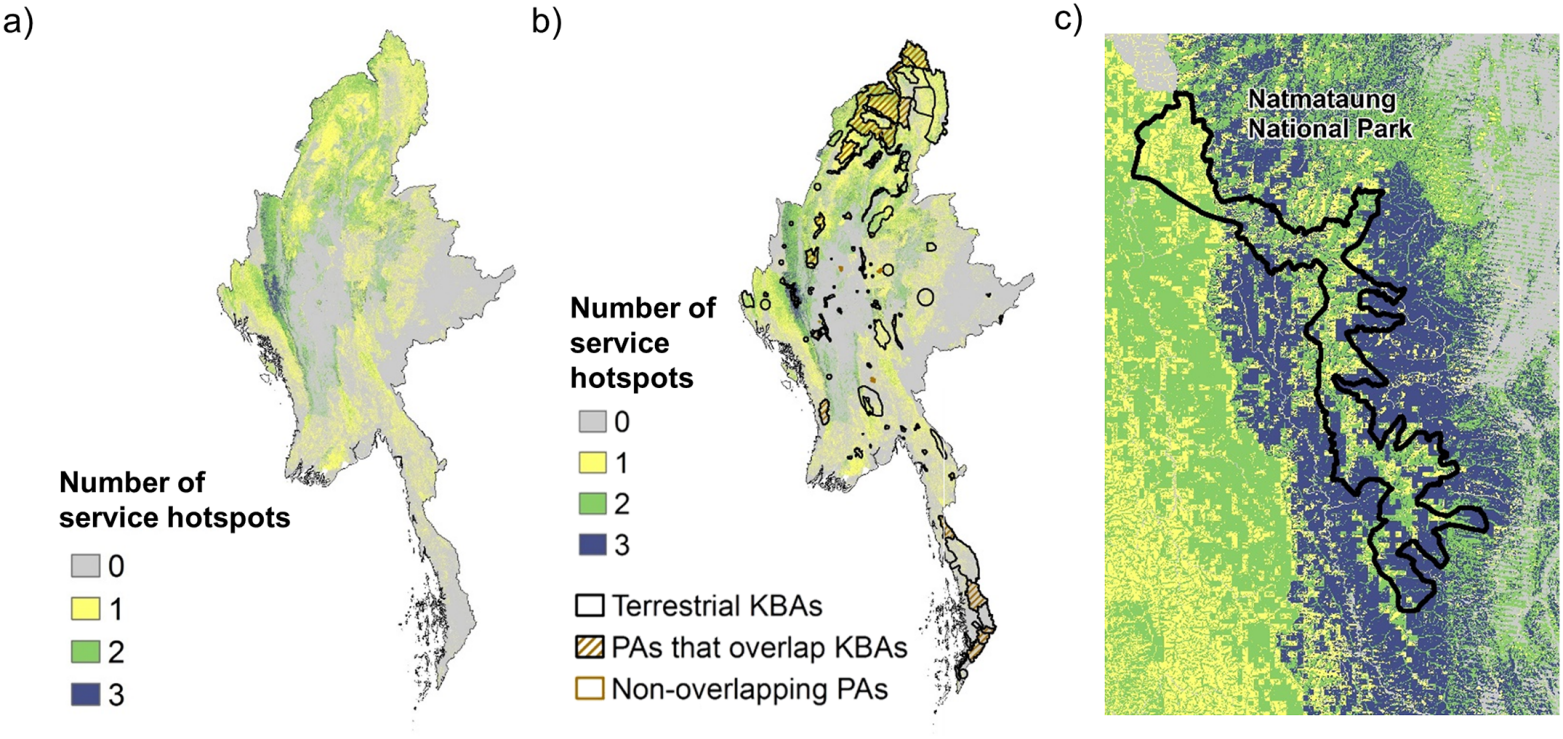
Overlap of ecosystem service provision hotspots (top 20% of service provision area) and their intersection with biodiversity conservation priority areas. The congruence of hotspots across all three services (a) is overlaid with terrestrial Key Biodiversity Areas (KBAs, open polygons) and protected areas (hatched polygons) (b); and zoomed into the highest-provisioning area to show detail (c). All maps are shown for baseline climate conditions.

Hotspots of current service provision (Fig 6) vary by service, but their overlap was greater than would be expected solely by chance. Across the three services considered, 1.9% of the country fell in the top 20% for all three services (vs. 0.8% expected if random overlap), 13.9% in the top 20% for two services (vs. 9.6% if random). Concordance between the hotspots for dry-season base flows for drinking water available and for inland flood risk reduction was the greatest, with 42% overlap. There was 32% overlap between hotspots for inland flood risk reduction and sediment retention for drinking water quality, and 25% overlap between dry season baseflows and sediment retention.

The designated KBAs cover 16.3% and existing PAs cover 7.4% of Myanmar’s land area, with over 99% of PAs also falling within a KBA. Overall, overlap between ES hotspots, KBAs and existing PAs was also greater than expected simply from chance (Fig 6): the area falling into the top 20% for at least one of the three services is 41.2% of Myanmar’s land area, but 50% of KBAs and 62.3% of PAs, representing 20% and 51% greater congruence than random. However, areas in the top 20% for all three services fell outside of KBAs or PAs more often than expected (8.1% vs 16.3% expected for KBAs, and 5.2% vs 7.4% expected for PAs). Twenty-nine percent of the sediment retention hotspot fell within KBAs, along with 25% of the inland flood risk reduction hotspot, but only 13% of the dry-season baseflow hotspot. PAs, which cover less than half the area of KBAs, included 17% of the inland flood risk reduction hotspot, 14% of the sediment retention hotspot and only 5% of the dry-season baseflow hotspot.

## Discussion

### Ecosystem service provision and susceptibility of beneficiaries to loss under baseline and future climate scenarios

Our results confirm that biophysical measures of ES supply alone are inadequate for determining the areas that deliver the greatest ES benefits to people or infrastructure (Fig 2, left column vs. right column). Instead, priority ES provision areas are shaped by variation in the spatial distribution of ES supply and demand, including variation in demand between beneficiary types (e.g., people dependent on surface water for drinking water vs. reservoirs). While the importance of considering both ES supply and demand has been widely acknowledged [52,53], consideration of the demand side of ES provision remains less common, particularly for regulating services like the ones we consider [10]. Given that a central objective of including ES information in development planning is to avoid harm to human well-being through avoiding harm to the environment, including the demand for ES in these processes is critical.

In addition, our results highlight two ways that climate change through the 2040s is likely to affect the role of natural ecosystems and the ES they provide. First, even if existing vegetation and land use remain unchanged, climate change may negatively affect water resources and increase flood risk. For example, under the high end of the climate change range, we found an increase in soil loss and higher transport of sediment to streams due to increases in the amount of precipitation. Although existing natural vegetation can buffer this process, it cannot fully mitigate it, as suggested by the difference in sediment export to reservoirs between the high end of the climate change range and baseline climate conditions, even in the absence of vegetation change.

Second, we find that the higher level of ES provided by natural vegetation under the high end of the climate change range is associated with increasing vulnerability of beneficiaries to vegetation loss. This suggests that the effects of climate change may be two-fold: both worsening environmental conditions and worsening the consequences of losing natural capital. To maintain baseline levels of water quality, water availability and flood risk likely requires more than preserving existing stocks of natural capital. Nature-based solutions, such as habitat restoration, can be considered alongside traditional, engineering-based approaches [54], and further assessments could help identify the most effective places for these activities. At the same time, it is important to recognize that necessary interventions to enhance ecosystems and their resilience can be challenging to effectively complete, and do not replace the need to protect existing natural capital [55]. Even with climate change likely to affect levels of ES provision, we find that the places providing the greatest ES benefits under baseline climate conditions are likely to remain important into the 2040s, under the range of climate change estimates we considered (Fig 5).

Our assessment therefore suggests that conservation of natural vegetation in these areas is likely to secure valuable benefits for the people of Myanmar today and in coming decades. We note that our analyses assume no major shifts in vegetation type or function occur over this time scale. Accounting for the effects of climate change on ES through changes in vegetation and ecological processes would be a valuable next step. Climate change effects could include direct ecological responses to changes in climate conditions, as well as indirect effects through human activities due to climate change, including land conversion and changes in agricultural practices. Detailed national-scale information on the effects of climate change on vegetation in Myanmar is lacking. However, global models suggest that Myanmar’s vulnerability to vegetation shifts from climate change is relatively low [56] and that the East Asian region is likely to experience a less than 10 percent change in vegetation type through 2050, the time period considered in our study [57]. Climate feedbacks, shocks, surprises, and extreme climate events not captured by the climate methods described here, which are much more difficult to model and predict, could also compromise the ability of these systems to deliver services at their full capacity.

Our results also highlight the importance of approaches to tackling uncertainty in planning for climate change impacts. The differences in ES provision between the two ends of the climate change range demonstrate this. This said, greenhouse gas emissions scenarios have proven to be historically conservative, with subsequent revisions toward expected higher emissions. With current global emissions rates tracking RCP 8.5, planning for the change in provision under this scenario would be the most conservative means of reducing risk.

### Spatial concordance across conservation objectives

Although we find that the most important areas for drinking water quality regulation, dry-season water availability and inland flood risk reduction overlap more than would be expected by chance, there is substantial variation due to differences in both the biophysical processes underlying service provision and the location of beneficiaries. Securing ample levels of these services for the people who rely on them will likely require management that targets different services separately. Our results mirror findings from elsewhere in the world [58–61] that suggest that biodiversity and ES priorities cannot substitute for each other due to the complex and varying relationships between them, but that there is great opportunity in directing conservation efforts to areas of synergy. In particular, ES priority areas that fall within KBAs but outside established protected areas could be managed to secure benefits to people and contribute to biodiversity conservation. Conservation of areas outside KBAs might also help secure connectivity among current conservation priority areas, as well as enhance the ability of species and ecosystems to adapt to future climate regimes [30].

### Implications for development planning in Myanmar

Our results provide a starting point for integrating ES information into national land use, development and climate adaptation planning in Myanmar. A number of national policies and strategies, including a Green Economy Strategy and Framework, Climate Change Strategy and Action Plan, Biodiversity Strategy and Action Plan and Land Use Policy, are currently being developed or have been already adopted. Information on ES will be key to ensure successful implementation of these policies and guide decision-making when it comes to land use and investments that can create economic, social and environmental benefits, including resilience to the impacts of climate change. By highlighting areas where loss of natural ecosystems would have outsized negative consequences for Myanmar’s people and existing infrastructure, the national assessment can guide land use planning for agricultural expansion and resettlement plans, as well as inform the location and design of infrastructure projects such as roads, transmission lines, canals and hydropower and other energy projects.

We have shared our results with a range of ministries involved in land use planning and other development and economic decision-making in Myanmar, and they are currently being used to advance ecosystem and natural capital accounting, as part of the reform process of the country’s national accounting systems. Natural capital and ecosystem services are now emphasized in the final draft National Environmental Policy of Myanmar, which is expected to adopted by the end of 2017. In collaboration with Myanmar’s Ministry of Natural Resource and Environmental Conservation (MoNREC), results are also available online to the public at www.myanmarnaturalcapital.org.

Because regional- and state-level land use planning and climate change adaptation planning will play an important role in Myanmar’s development trajectory, refining and updating this national assessment to provide information relevant to decision-making at these levels will also be important. This is especially critical given that national ES priorities based on total population are heavily influenced by the country’s largest urban centers and may not reflect regional priorities or the needs of rural populations [62]. Achieving equitable, inclusive development requires consideration of how the value or importance of ES from a given area varies among beneficiary groups, even for the same set of biophysical processes [44,63]. Accounting for specificities in regional demand will be an important addition. For example, for the dry-season baseflow whose demand is driven by households relying on surface water, we did not account for current or future regional water scarcity: in wet regions where dry-season baseflow is sufficient for local needs, demand for the baseflow regulation service may be very low.

Finally, assessment of additional ES, and their response to land use change and climate change, would be a valuable step for future work, at both the national and sub-national levels. The services assessed here were particularly limited by the availability of spatial data, both ecological and socio-economic. The societal and economic value of many other ES such as non-timber forest products, nursery and breeding habitat contributing to fisheries, and wild insect pollination contributing to crop production are likely quite high [64], but a lack of comprehensive data prevents a detailed understanding of how production of services varies spatially, where ES benefits flow to, and how the value might change with forest loss or climate change.

In interpreting the maps provided in this study, several points should be noted. First, the outputs of the biophysical models are best used to assess relative spatial or temporal differences. Given that the uncertainties around future estimates of climate change have large effects on biophysical predictions, a robust interpretation of results consists in identifying areas that are consistently identified as hotspots of services. Second, the weighting approach used to combine ES supply and demand is subject to limitations of multi-criteria analyses [65,66]. Finally, we assess ES benefits by comparing existing LULC to a scenario in which natural vegetation was converted to agriculture. While the alternate scenario reflects national trends, refining these scenarios in order to explore likely or potential futures at the regional level would provide an opportunity to increase the relevance of our results to decision making.

### Future directions for integrating natural capital assessments and climate change scenarios to inform development planning

The current decade has seen growing attention to the impacts of climate change on ES. However, existing studies are frequently disconnected from decision-making contexts and only rarely assess the robustness of decision-making outcomes such as policies or management strategies to sources of uncertainty, including climate change scenarios [11]. The integration of ES assessments and climate change projections in decision-relevant ways is an important frontier.

In the context of development planning, delivering assessments on policy-relevant temporal and spatial scales that can guide decisions that will be robust to climate change is critical. This is particularly important in rapidly developing countries where the stakes are highest both for human well-being and biodiversity. Towards this goal and based on our experiences developing a national assessment for Myanmar, we suggest several directions for furthering the integration of natural capital assessments and climate change scenarios.

Advances in data and modeling would provide a more precise and nuanced understanding of future changes in ES provision with climate change. In particular, future assessments would benefit from: i) assessing the impacts of climate change on ES through changes in vegetation and land management; ii) accounting for the impacts of extreme weather events on natural capital and ES; iii) better baseline data to improve projections; and iv) improved accounting for and communication of uncertainty. Integration of climate information into ES assessments would be enhanced by the capacity to model a greater diversity of linkages between climate, ecosystems and ES. The ability to assess impacts of climate change on ES through changes in vegetation and land management, as previously discussed, as well as improved accounting for the impacts of extreme weather events, rather than relying on annual or monthly climate data, would be especially valuable. These efforts would benefit from more comprehensive observed baseline climate and land use data, requiring investment, integration of local expert knowledge, local capacity building, and collaboration across countries and sectors. Finally, in line with recognized challenges for ES assessments in general [67], a more complete accounting for uncertainty in ES outcomes would increase the robustness of assessment results. This includes uncertainty inherent within the InVEST models as well as originating in the data used, including both biophysical factors governing ES supply and the demographic, socio-economic, political and institutional factors governing demand for and access to ES benefits.

Our assessment process also highlighted trade-offs among analysis complexity, knowledge ownership by stakeholders and decision-making relevance. More sophisticated, dynamic models would provide a more precise characterization of the effects of climate change on natural capital and ES provision. However, this inherently increases data requirements and analytical complexity, resulting in proportionate difficulty in developing local capacity to replicate and build on assessments, potentially affecting the impact of assessments on planning decisions. Balancing this trade-off between complexity, feasibility and utility can be difficult, and requires consideration of the ultimate aims of producing integrated ES and climate assessments: is it to provide a foundation for locally-based knowledge production, shape stakeholder thinking, inform a specific policy or decision, or some combination? Different answers require different approaches [68] and may be best achieved by focusing on different aspects of the knowledge production process [69].

As our experiences here illustrate, however, even under conditions of limited data and rapid timelines, it is possible to produce initial, policy-relevant assessments of natural capital and ES benefits to people and infrastructure under future climate scenarios that can set the stage for decision-making and more in-depth research.

## Supporting information

S1 Appendix InVEST model details and inputs. (DOCX)

S2 Appendix LULC mapping methods. (DOCX)

S1 Data LULC map. (ZIP)

S2 Data Baseline climate data. (ZIP)

S3 Data 25th percentile RCP 4.5 climate data. (ZIP)

S4 Data 75th percentile RCP 8.5 climate data. (ZIP)
