# Supplementary material for: Assessing ecosystem service provision under climate change to support conservation and development planning in Myanmar
Source: PLoS One. 2017 Sep 21;12(9):e0184951. doi: 10.1371/journal.pone.0184951 (PMC5608473; doi:10.1371/journal.pone.0184951)
Supplement: S1 Appendix — (DOCX) [file pone.0184951.s001.docx]

**S1 Appendix: InVEST model parameterization and interpretation**

This appendix provides details on the input data, underlying assumptions, and interpretation of outputs for the three InVEST models used in the study. A full description of the models is available in Sharp *et al.* (2016), and additional details about the parameters and analyses in Myanmar are available in Wolny *et al.* (2016).

1. **Sediment delivery model**

The InVEST Sediment delivery (SDR) model is a simplified method for estimating the amount of soil that is eroded from the landscape, the amount that reaches a waterway, and the areas on the landscape that provide the service of retaining sediment. Table A.1 summarizes the inputs used in this study. Model results were output at 90m resolution.

**Table A.1:** Inputs used in the InVEST sediment delivery model

| **SDR model input** | **Dataset source** |
| --- | --- |
| Land use/land cover^*^ | Custom map made with Google Earth Engine, 150m resolution (see *Appendix B*); agriculture specified by administrative district based on FAO (2005) |
| Digital elevation model | USGS (2004), 90m resolution |
| Rainfall erosivity^+^ | Derived from precipitation data using the equation R = 38.5 + 0.35 P where R is rainfall erosivity and P is annual mean precipitation (mm/year); from Thang *et al.* (2005). Historical and future climate scenario precipitation data from downscaled climate modeling (see *Methods* section in main text). |
| Soil erodibility | Derived from the Harmonized World Soil Database (FAO/IIASA/ISRIC/ISS-CAS/JRC, 2012) using information on sand/silt/clay/organic carbon |
| Threshold flow accumulation | Value: 10000 (to define more major streams) |
| USLE coefficients | Based on a variety of literature sources, primarily Merritt (2002).  USLE P was kept constant, as no information on erosion control practices was available nationally.  USLE C values were as follows:  Forest 0.02  Open forest 0.02  Scrubland 0.048  Mangrove 0  Water 0  Snow 0  Agriculture, defined by district Varies (see below) |

^*^Input varies between land use scenarios

^+^Input varies between climate scenarios

To derive agriculture values by district crop type, the original single “Agriculture” LULC class was divided by district boundaries. This produces one distinct agriculture LULC class per district. Crop information for each district came from the [*Digital Agricultural Atlas of the Union of Myanmar*](http://dwms.fao.org/atlases/myanmar/index_en.htm) (FAO, 2005), which provides district boundaries, the types of crops grown in each district, and the amount of area in the district that the crop is grown on. For each crop type grown in a district, a USLE C value was assigned, then weighted by the percent of the total agricultural area that crop is grown in. The resulting weighted USLE C values were added together for all crops grown in the district to calculate the final value used in the coefficient table. This provides differentiation across the country, based on differences in agriculture, that a single agriculture LULC class for the whole country does not.

A second, hypothetical, LULC map was also created, where all land in the country was converted to agriculture. This was done according to district, as described above.

*Interpreting outputs*

The main result from the model is sediment export, which represents the amount of sediment that erodes from the landscape and is not retained by downslope vegetation on its path to the stream. Sediment export is calculated using the Universal Soil Loss Equation (USLE) and Sediment Delivery Ratio (SDR) methods. Sediment retention results show the difference in sediment export between the all-agriculture and baseline LULC maps. These analyses were by necessity restricted to Myanmar, which means that contributions from areas of watersheds outside the country’s boundaries were ignored.

Due to the scarcity of data and the limited resources available for this study, no model calibration was attempted. Absolute predictions of sediment export should be interpreted with care, but recent work suggests that application for relative predictions, i.e. the maps that are used to compute service provision, was not significantly affected by calibration (Hamel et al. 2015). Another limitation of the model includes the exclusion of other sources of sediment (e.g. landslides, gully), as explained by Hamel et al. (2015), which are arguably less affected by changes in natural ecosystems.

1. **Seasonal water yield model**

We used the InVEST seasonal water yield (SWY) model version 3.3.0, which computes three indices: quickflow (QF), local recharge (R), and baseflow (B), which are all based on monthly climate values. In this study, only quickflow and baseflow are used. Quickflow represents the amount of precipitation that is converted to direct runoff, entering streams soon after a rain event, and is computed based on the curve number methods (NRCS-USDA, 2004b). Baseflow represents the amount of precipitation that enters streams through subsurface flow, both during and in-between rain events. This approach requires monthly precipitation values to be disaggregated on an event basis, following the method described by Guswa et al. (2017). Event contributions are then summed up to provide an annual average, expressed in mm. Table A.2 summarizes the inputs used in the InVEST seasonal water yield model. Model results were output at 150m resolution.

**Table A.2:** Inputs used in the InVEST seasonal water yield model

| **SWY model input** | **Dataset source** |
| --- | --- |
| Precipitation^+^ | Amount and number of rain events >0.2 mm per month. Historical and future climate scenario precipitation data from downscaled climate modeling (see *Methods* section in main text). |
| Reference evapotranspiration^+^ | Derived from historical and future climate scenarios of precipitation and temperature data (see *Methods* section in main text), using the Modified Hargreaves method (Droogers & Allen, 2002). |
| Digital Elevation Model | USGS (2004), 90m DEM, resampled to 150m |
| Land use/land cover map^*^ | Custom map made with Google Earth Engine, 150m resolution (see *Appendix B*); agriculture specified by administrative district based on FAO (2005) |
| Soil group | HiHydroSoil (De Boer, 2015) |
| Are of interest/watershed | Polygon outline of the country of Myanmar (Note that this is not hydrologically complete where watersheds cross the country border.) |
| Climate zones | Created Thiessen polygons around the climate data points (latitude/longitude values) from the downscaled climate modeling (see *Methods* section in main text). |
| Climate zone table^+^ | Number of rainy days per month, for historical and future climate scenarios from downscaled climate modeling (see *Methods* section in main text). |
| Biophysical table | Values from a variety of literature sources. Crop coefficients (Kc) primarily from Allen et al. (1998). Curve number (CN) values primarily from NRCS-USDA (2004a).   \| **LULC description** \| **Kc_1^1^** \| **CN_A** \| **CN_B** \| **CN_C** \| **CN_D** \| \| --- \| --- \| --- \| --- \| --- \| --- \|  \| Forest \| 1 \| 36 \| 60 \| 73 \| 79 \| \| --- \| --- \| --- \| --- \| --- \| --- \| \| Open forest \| 0.9 \| 43 \| 65 \| 76 \| 82 \| \| Scrubland \| 0.43 \| 35 \| 56 \| 70 \| 77 \| \| Mangrove \| 1.1 \| 98 \| 98 \| 98 \| 98 \| \| Water \| 1.05 \| 1 \| 1 \| 1 \| 1 \| \| Snow \| 0.4 \| 1 \| 1 \| 1 \| 1 \|  \| Agriculture, defined by district \| Varies^2^ \| Varies^2^ \| Varies^2^ \| Varies^2^ \| Varies^2^ \| \| --- \| --- \| --- \| --- \| --- \| --- \| |
| α, β, γ | Default parameters: (1/12, 1,1) |

^*^Input varies between land use scenarios

^+^Input varies between climate scenarios

^1^This is the crop factor for month 1 (January.) Kc is constant across the year except for crops.

^2^As with the USLE C coefficients, Kc and CN were calculated for each district’s mix of agriculture, using a similar method. Monthly values of Kc are used in the SWY model. We obtained information on seasonal crop growth from the *Digital Agricultural Atlas of the Union of Myanmar* (FAO, 2005). For this analysis, ‘monsoon’ months were taken as May through October, ‘summer’ months March and April, and ‘winter’ months November through February. This seasonal information was used to determine monthly Kc values, based on the percent area that the crop is grown in per district, and Kc(ini), Kc(mid) and Kc(end) values from Allen *et al.* (1998). Similar to the USLE C parameter, Kc and CN values per crop were weighted by the percent of the district’s agricultural area that crop is grown in, and the weighted values added together, to produce the final coefficient values for each district.

*Interpreting outputs*

*Flow retention*

The flow retention index, used as a proxy for flood risk reduction, was defined as:

Flow retention=1-QF/P

where QF is the sum of quickflow over the monsoon months (June –September) and P is total precipitation over those same months. The index ranges between 0 and 1; a value of 0 corresponds to no retention by the pixel, a value of 1 corresponds to total retention. Importantly, it does not include information on downslope or upslope areas, which drastically simplifies hydrologic processes since runoff produced in upslope areas can be attenuated before reaching the stream. More detailed analyses based on information about past floods and a more sophisticated modeling approach could be performed for areas of interest. However, this index captures an important driver of flooding, the production of surface runoff, based on a commonly-used hydrologic model, which suggests that the approach is adequate for the screening-level analyses presented here.

*Dry-season flow*

In the absence of detailed hydrogeological information in the country (see review by McCartney et al. 2013), the three model parameters that determine baseflow processes were set to their default values (Table A.2). This assumption means that, for each pixel, one twelfth of the upslope subsidy, i.e., the water recharged by upslope areas, is available each month for evapotranspiration. In reality, higher or lower amounts of water may be available depending on subsurface flow rates, and the local soil and bedrock properties.

To assess the implications of this assumption, we examined the local recharge index computed by the model, i.e., the net recharge on each pixel. Only 0.5% of the pixels had negative values, meaning that the local water balance did not use upslope subsidies for >99% of the pixels. In these cases, the constraint on use of upslope subsidies (one twelfth annual recharge) is not used by the model; rather, the water balance is constrained by the energy demand (potential evapotranspiration). Correlation analyses also confirmed that baseflow indices were strongly influenced by precipitation and LULC, which suggests that the index captures the main processes driving baseflow recharge (precipitation and partitioning between infiltration and surface runoff).

Of note, by comparing baseflow index values between scenarios (LULC and climate), in order to obtain the difference maps in Figures 2, 3, and 4, we used absolute values of the baseflow index. However, as noted in the InVEST user’s guide, the pixel-level index values provide information on spatial trends in baseflow provisioning, and their absolute values should be interpreted with caution. This may explain some negative values of the “contribution to baseflow service” maps (Figure 2) and explains the relative scale we used for these maps.

As with the sediment retention modeling, these analyses were restricted to Myanmar, ignoring contributions from areas of watersheds beyond the country’s boundaries.

1. **Coastal vulnerability model**

The InVEST coastal vulnerability (CV) model produces a qualitative estimate of how changes to natural habitats can affect coastal communities’ exposure to storm-induced erosion and flooding. By considering biological and geophysical factors along the coastline, the model differentiates areas with relatively high or low exposure to erosion and inundation during storms, and indicates the role that natural habitats play in helping reduce that exposure. Combining these results with global population information can show areas along a given coastline where humans are most vulnerable to storm waves and surge, and where natural habitats play the greatest role in protecting people. Table A3 summarizes the inputs used in the InVEST coastal vulnerability model.

**Table A.3:** Inputs used in the InVEST coastal vulnerability model

| **CV model input** | **Dataset source** |
| --- | --- |
| Area of interest | Drawn by hand to include the coast of Myanmar and the extent of sea level rise point data from downscaled climate modeling (see *Methods* section in main text). |
| Land polygon | Global land polygon provided in the InVEST sample data |
| Bathymetry layer | GEBCO Bathymetry (GEBCO 2014) |
| Relief | USGS HydroSHEDS (Lehner et al. 2008) |
| Natural habitats | Locations of coral reefs, sea grass and mangroves from UNEP-WCMC (Giri et al. 2011, UNEP-WCMC 2016, UNEP-WCMC 2010). Continental mangrove data for Tanintharyi was updated with forest mapping data from MOECAF. The associated “natural habitats table” is shown below. |
| Climatic forcing grid | WAVEWATCH III (WW3DG, 2016), processed by the Natural Capital project and provided in the InVEST sample data |
| Continental shelf | Continental Shelf polygon provided in the InVEST sample data |
| Sea level rise^+^ | Generated from latitude/longitude and sea level rise data for future climate scenarios based on downscaled climate modeling (see *Methods* section in main text). |
| Population layer | WorldPop gridded population (Gaughan *et al.*, 2013) |
| Natural habitats table | The RANK field gives a relative ranking of how much protection each habitat provides for the coast – a value of 1 indicates highest protection, 4 lowest. PROTECTION DISTANCE indicates the distance over which the habitat has a protective influence, given in meters. The values assigned were based on general global guidelines and would benefit from more detailed study.   \| **HABITAT** \| **ID** \| **RANK** \| **PROTECTION DISTANCE** \| \| --- \| --- \| --- \| --- \| \| mangroves \| 1 \| 1 \| 2000 \| \| coralreefs \| 2 \| 1 \| 2000 \| \| seagrass \| 3 \| 4 \| 2000 \| |

^+^Input varies between climate scenarios

*Interpreting outputs*

The main output from the model contains information on the different factors related to coastal exposure, and how they impact each shoreline segment. It also contains a “habitat role” index, indicating how much protection coastal natural habitat provides to that shoreline segment, which is the result that was used in this analysis. All values are indices, relative to each shoreline segment in the modeled area of interest.

To create the final service map, population density for each shoreline segment was multiplied by the “habitat role” index. This provides an indication of where coastal natural habitats provide the greatest protection to the greatest number of people.

The results of the CV model cannot be directly compared between scenarios, as the high/low values are relative within each scenario. Post-processing of the results (using custom geoprocessing scripts) was done to allow comparison between sea level rise scenarios, which showed that the same places along the coast where habitats play the greatest role now, also play the greatest role later.

**References**

Allen, R.G., Pereira, L.S., Raes, D., Smith, M., 1998. Crop evapotranspiration - Guidelines for computing crop water requirements, FAO Irrigation and drainage paper 56. Rome, Italy.

De Boer, F., 2015. HiHydroSoil: A High Resolution Soil Map of Hydraulic Properties. Report 134; Available online at: [www.futurewater.nl](http://www.futurewater.nl).

Droogers P., Allen R.G., 2002. Estimating reference evapotranspiration under. Irrigation and drainage systems*.* 16, 33–45. doi: 10.1023/A:1015508322413

FAO (Food and Agricultural Organization of the United Nations), 2005. Digital Agricultural Atlas of the Union of Myanmar. FAO, Rome. Available online: <http://dwms.fao.org/atlases/myanmar/index_en.htm>

FAO/IIASA/ISRIC/ISS-CAS/JRC (2012), *Harmonized World Soil Database (ver. 1.2)*, FAO, Rome, Italy. Available at: [www.fao.org/soils-portal/soil-survey/soil-maps-and-databases/harmonized-world-soil-database-v12/en/](http://www.fao.org/soils-portal/soil-survey/soil-maps-and-databases/harmonized-world-soil-database-v12/en/)

Gaughan A.E., Stevens F.R., Linard C., Jia P., Tatem A.J., 2013. High resolution population distribution maps for Southeast Asia in 2010 and 2015. PLoS ONE. 8(2), e55882. <http://www.worldpop.org.uk/>.

GEBCO (General Bathymetric Chart of the Oceans), 2014. GEBCO_2014 Grid. Available online at: <http://www.gebco.net>.

Giri C., Ochieng E., Tieszen L.L., Zhu Z., Singh A., Loveland T., Masek J., Duke N., 2011. Status and distribution of mangrove forests of the world using earth observation satellite data (version 1.3, updated by UNEP-WCMC). Global Ecology and Biogeography. 20, 154–159. doi: [10.1111/j.1466-8238.2010.00584.x](http://dx.doi.org/10.1111/j.1466-8238.2010.00584.x) . Available online at: <http://data.unep-wcmc.org/datasets/4>.

Guswa, A. J., Hamel, P., & Meyer, K. (in press). Curve number approach to estimate monthly and annual direct runoff. Journal of Hydrologic Engineering.

Hamel, P., Chaplin-Kramer, R., Sim, S., Mueller, C., 2015. A new approach to modeling the sediment retention service (InVEST 3.0): Case study of the Cape Fear catchment, North Carolina. Science of the Total Environment. 524–525.

Lehner, B., Verdin, K., Jarvis, A., 2008. New global hydrography derived from spaceborne elevation data. Eos, Transactions, AGU. 89(10), 93–94. Available online at: [http://hydrosheds.cr.usgs.gov](http://hydrosheds.cr.usgs.gov/).

McCartney, M., Pavel, P., Latt, K., Zan, K., Thein, K., 2013. Water Resource Assessment of the Dry Zone of Myanmar: Final Report for Component 1.

Merritt, W., 2002. Biophysical Considerations in Integrated Catchment Management: A Modelling System for Northern Thailand. Thesis, Australian National University.

NRCS-USDA. 2004a. Chapter 9. Hydrologic Soil-Cover Complexes. United States Department of Agriculture, Part 630 Hydrology. National Engineering Handbook. United States Department of Agriculture

NRCS-USDA, 2004b. Chapter 10. Estimation of Direct Runoff from Storm Rainfall. In: United States Department of Agriculture, Part 630 Hydrology. National Engineering Handbook. United States Department of Agriculture.

Sharp, R., Tallis, H.T., Ricketts, T., Guerry, A.D., Wood, S.A., Chaplin-Kramer, R., Nelson, E., Ennaanay, D., Wolny, S., Olwero, N., Vigerstol, K., Pennington, D., Mendoza, G., Aukema, J., Foster, J., Forrest, J., Cameron, D., Arkema, K., Lonsdorf, E., Kennedy, C., Verutes, G., Kim, C.K., Guannel, G., Papenfus, M., Toft, J., Marsik, M., Bernhardt, J., Griffin, R., Glowinski, K., Chaumont, N., Perelman, A., Lacayo, M. Mandle, L., Hamel, P., Vogl, A.L., Rogers, L., Bierbower, W., 2016. InVEST 3.3.0 User’s Guide. The Natural Capital Project, Stanford University, University of Minnesota, The Nature Conservancy, and World Wildlife Fund.

Thang, C.C., Euimnoh, A., Shivakoti, G.P., Clemente, R., 2005. Spatial modeling for land degradation assessment using remotely sensed data and geographic information system: a case study of Daungnay Watershed, Magway District, Myanmar. In: Conference Proceedings: Map Asia, 2005.

WW3DG (The WAVEWATCH III Development Group), 2016: User manual and system documentation of WAVEWATCH III version 5.16. Tech. Note 329, NOAA/NWS/NCEP/MMAB, College Park, MD, USA, 326 pp.+ Appendices.  <http://polar.ncep.noaa.gov/waves/>.

UNEP-WCMC, Short F.T., 2016. Global distribution of seagrasses (version 4.0). Fourth update to the data layer used in Green and Short (2003). Cambridge (UK): UNEP World Conservation Monitoring Centre. Available online at: <http://data.unep-wcmc.org/datasets/7>.

UNEP-WCMC, WorldFish Centre, WRI, TNC, 2010. Global distribution of warm-water coral reefs, compiled from multiple sources including the Millennium Coral Reef Mapping Project. Version 1.3. Includes contributions from IMaRS-USF and IRD (2005), IMaRS-USF (2005) and Spalding et al. (2001). Cambridge (UK): UNEP World Conservation Monitoring Centre. Available online at: <http://data.unep-wcmc.org/datasets/1>.

USGS (United States Geological Survey). 2004. Shuttle Radar Topography Mission, 1 Arc Second scene SRTM_u03_n008e004, Unfilled Unfinished 2.0, Global Land Cover Facility, University of Maryland, College Park, Maryland, February 2000

Wolny S., Hamel, P., Mandle, L., 2016. Myanmar national ecosystem service assessment technical report. Natural Capital Project, Stanford University.
