# Supplementary material for: Assessing ecosystem service provision under climate change to support conservation and development planning in Myanmar
Source: PLoS One. 2017 Sep 21;12(9):e0184951. doi: 10.1371/journal.pone.0184951 (PMC5608473; doi:10.1371/journal.pone.0184951)
Supplement: S2 Appendix — (DOCX) [file pone.0184951.s002.docx]

**S2 Appendix. Myanmar 2013-2014 land cover dataset processing**

A land cover dataset (Fig. 1) was created with Google Earth Engine (GEE) to complete national assessments of natural capital in Myanmar. The new land cover dataset was needed due to the absence of a land cover map with better accuracy than Globcover 2009 published in peer-reviewed literature (Bontemps et al., 2011). The GEE national dataset was thus created to provide better land cover information, with higher accuracy and more resolution, than is currently available elsewhere. Analysis resulting from this dataset should be interpreted with caution and the caveat that more accurate land cover information should be integrated as soon as it becomes available. Of note, plantations are not differentiated in this land cover dataset, and at current are likely classified within the open and closed forest classes. Plantations present an exceptional challenge in differentiating their spectral signature due to similarity with naturally occurring trees.

*Method*

GEE provides a graphical interface for users to complete a supervised land cover classification. Google’s satellite imagery base layer was used to apply points and polygons that the classifying algorithm used to identify areas of similar spectral reflectance. The use of the satellite base layer is problematic due to the unknown date of imagery acquisition. Because of this uncertainty, large contiguous areas of high spectral similarity (like large forest blocks) were used for training points and polygons. Boundary areas were avoided. Approximately 25 points and 10 polygons were created for each land cover class. The Naïve Bayes classification algorithm was used to produce the classification. The resolution of the classification was set to 150 meters.

The ‘Landsat TOA Percentile Composite’ data product, set to the Landsat 7 collection between the dates of 11 November 2013 to 10 June 2014, was used as source imagery. The dates were chosen as a rough beginning and end of the dry season in Myanmar. The Landsat TOA Percentile Composite is a product that assigns pixels a darkness and lightness percentage. Pixels closest to the 50^th^ percentile were chosen to minimize clouds and shadows.

A set of land cover classes was developed with information from personal communications with mid-level technicians in Myanmar’s Ministry of Environmental Conservation and Forestry (MOECAF) during a workshop in June of 2014. These are detailed in Table 1 with definitions. Definitions for each land cover class were developed ad hoc.


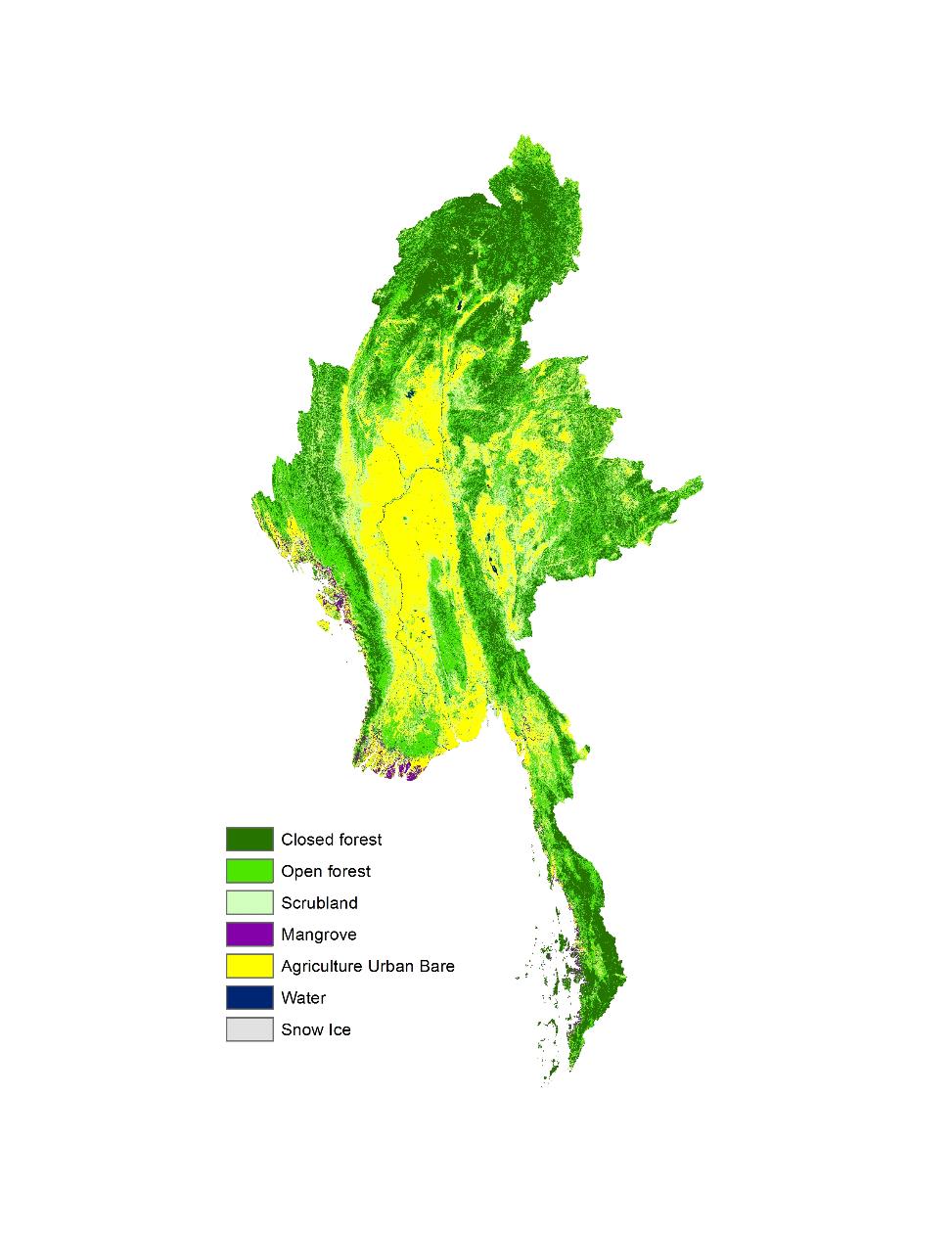


**Figure 1.** GEE 2013-2014 Myanmar land cover

**Table 1.** Land cover classes with definitions

| **Land cover class** | **Definition** |
| --- | --- |
| 1 | ***Closed forest*** – closed tree canopy, greater than 80% cover |
| 2 | ***Open forest*** - open tree canopy, between 20 and 80% cover |
| 3 | ***Scrubland*** – sparse trees and shrubs with herbaceous layer, some bare rock and soil |
| 4 | ***Mangrove*** – coastal tree cover, often with water pixels intermixed |
| 5 | ***Agriculture/urban/bare*** – row crop agriculture, including rice paddy, barren land, impervious surfaces including built surfaces |
| 6 | ***Water*** – surface water body |
| 7 | ***Snow and ice*** – snow and ice located in high elevation mountain ranges |

The land cover classification was created in a series of steps in GEE and then exported and further processed in ArcGIS for Desktop 10.3.1 using map algebra. The mangrove and closed forest classes were difficult for the supervised classification algorithm to distinguish, and so were modified using map algebra.. For instance, the mangrove class was frequently misclassified outside of coastal zones. However, the classifier in GEE appeared to adequately differentiate between dryland forests and mangroves when the analysis was completed at a more local scale, and so mangrove was classified independently, then integrated into the dataset using reclassifications and map algebra in ArcGIS.Another difficultly for the classifier was the presence of shadows due to hillshade in the mountainous north of the country. The classifier originally misclassified many areas in hillshade as water pixels. These were manually removed by creating a forest mask in the northern mountainous regions and again using a series reclassifications and map algebra in ArcGIS.

A set of 384 points were randomly generated across the country posteriori of the analysis. Each point was validated against high resolution imagery available in Google Earth. Five points were dropped due to cloud cover, leaving the total number of points 379. The total number of points per land cover class are detailed in Table 2. Snow and Ice were not included in the accuracy assessment due to the small amount of actual land area.

**Table 2.** Total number of accuracy assessment points per class

| **Land cover class** | **Number of points** |
| --- | --- |
| Agriculture/urban/bare | 83 |
| Closed forest | 104 |
| Mangrove | 2 |
| Open forest | 118 |
| Scrubland | 66 |
| Water | 6 |

The total accuracy of the map based on this technique was found to be 85%, with a Kappa statistic of 80%. These values provide assurance that the land cover map is of high enough accuracy to be used in a national scale assessment of ecosystem services. The results of the accuracy assessment were expected due to the scale at which the land cover classification was completed (150 m) and the small number of classes used. A confusion matrix is detailed in Table 2, with user’s and producer’s accuracy reported. Most classes report above 80% user’s and producer’s accuracy. The exception to this is the user’s accuracy for the scrubland class which reported at just 50%. This class was found to be spectrally similar to the Agriculture/urban/bare class and was often misclassified as such.

**Table 3.** Confusion matrix for accuracy assessment

|  | Agriculture/urban/bare | Closed forest | Open forest | Mangrove | Scrubland | Water | Total | User's accuracy |
| --- | --- | --- | --- | --- | --- | --- | --- | --- |
| Agriculture/urban/bare | 76 | 1 | 2 | 0 | 3 | 1 | 83 | 0.92 |
| Closed forest | 0 | 99 | 4 | 0 | 1 | 0 | 104 | 0.84 |
| Open forest | 3 | 5 | 108 | 0 | 0 | 2 | 118 | 0.92 |
| Mangrove | 0 | 0 | 0 | 2 | 0 | 0 | 2 | 1.00 |
| Scrubland | 10 | 1 | 22 | 0 | 33 | 0 | 66 | 0.50 |
| Water | 0 | 0 | 0 | 0 | 0 | 6 | 6 | 1.00 |
| Total | 89 | 106 | 136 | 2 | 37 | 9 | 379 | 0.862 |
| Producer's accuracy | 0.85 | 0.93 | 0.79 | 1.00 | 0.89 | 0.67 | 0.857 |  |
|  |  |  |  |  | Total accuracy | | | 0.855 |
|  |  |  |  |  | Kappa statistic | | | 0.805 |

*Reference*

Bontemps S., Defourny P., Bogaert E., Arino O., Kalogirou V., & Perez J. (2011) GLOBCOVER 2009 - Products description and validation report.
