# Supplementary figures and images for: Assessing ecosystem service provision under climate change to support conservation and development planning in Myanmar
Source: PLoS One. 2017 Sep 21;12(9):e0184951. doi: 10.1371/journal.pone.0184951 (PMC5608473; doi:10.1371/journal.pone.0184951)

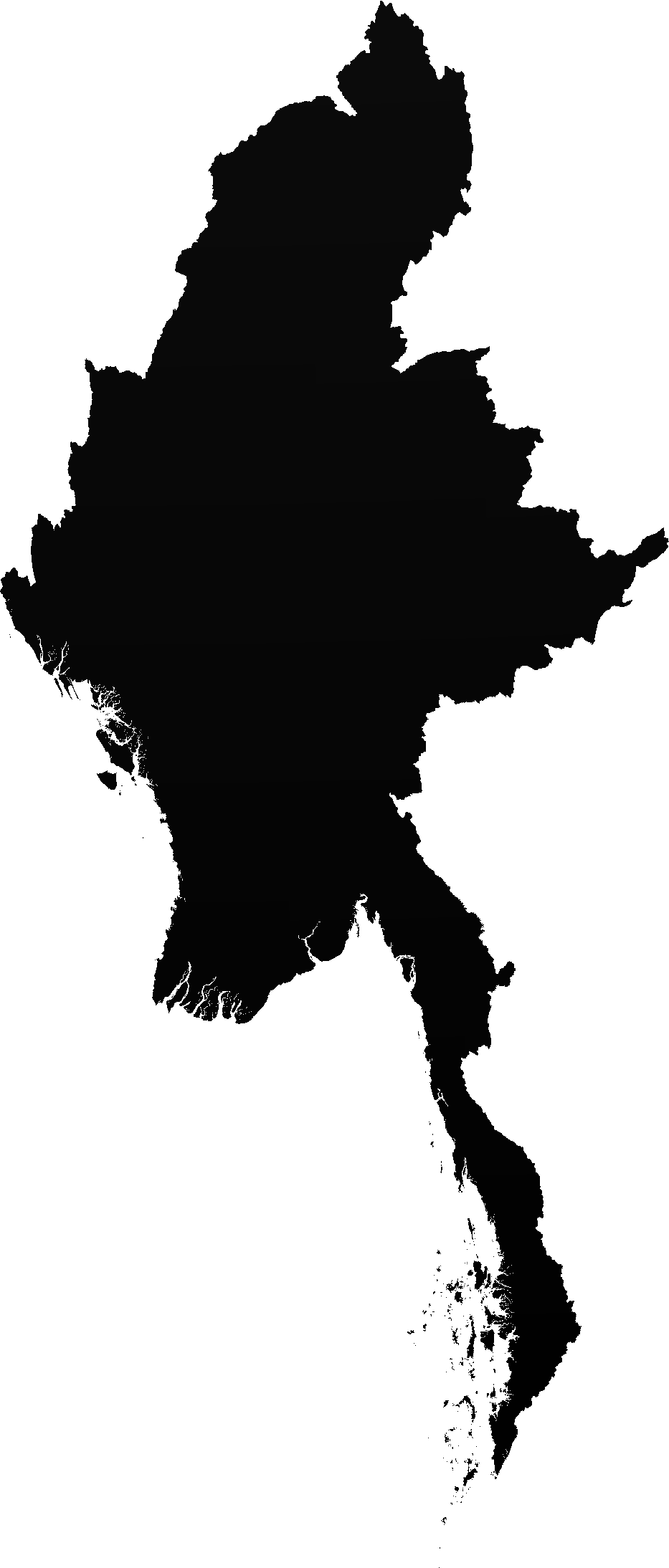

Supplement: S2 Data — (ZIP) [file pone.0184951.s004.zip › Myanmar_climate_data_historical/climate_zones_my.tif]

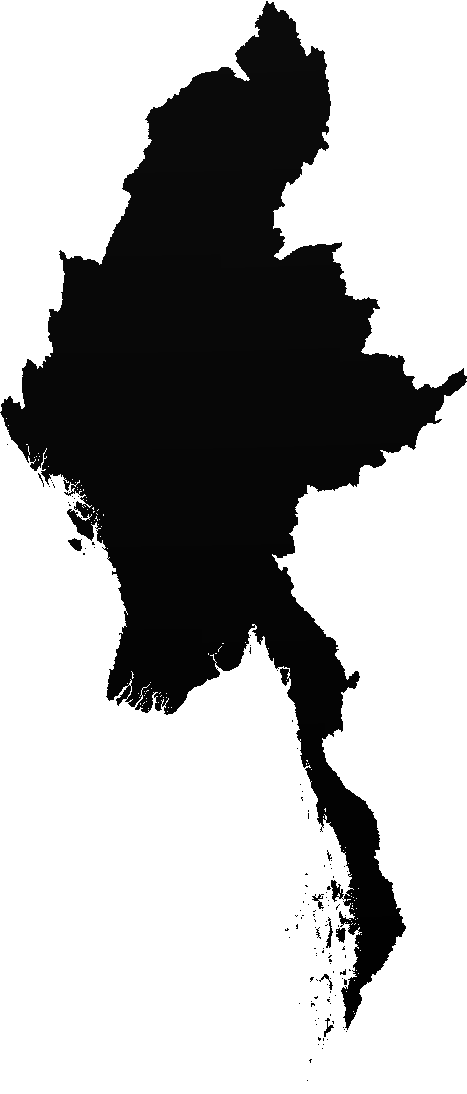

Supplement: S2 Data — (ZIP) [file pone.0184951.s004.zip › Myanmar_climate_data_historical/climate_zones_my.tif.ovr]
